# Supplementary material for: Ligand-modified nanoparticle surfaces influence CO electroreduction selectivity
Source: Nat Commun. 2024 Apr 6;15:2995. doi: 10.1038/s41467-024-47319-z (PMC10998913; doi:10.1038/s41467-024-47319-z)
Supplement: Supplementary file 1 — Supplementary Information [file 41467_2024_47319_MOESM1_ESM.pdf]

Supplementary Information

Ligand-modified Nanoparticle Surfaces Influence CO Electroreduction Selectivity

Erfan Shirzadi[1,9], Qiu Jin[2,9], Ali Shayesteh Zeraati[3], Roham Dorakhan[1], Tiago J. Goncalves[2], Jehad Abed[1,4], Byoung-Hoon Lee[1], Armin Sedighian Rasouli[1], Joshua Wicks[1], Jinqiang Zhang[1], Pengfei Ou[1], Victor Boureau[5], Sungjin Park[1], Weiyan Ni[1], Geonhui Lee[1], Cong Tian[1], Debora Motta Meira[6,7], David Sinton[3], Samira Siahrostami[8]*, and Edward H. Sargent[1]*

[1]Department of Electrical and Computer Engineering, University of Toronto, Toronto, Ontario, Canada.

[2]Department of Chemistry, University of Calgary, 2500 Calgary, Alberta, Canada.

[3]Department of Mechanical and Industrial Engineering, University of Toronto, Toronto, Ontario, Canada.

[4]Department of Materials Science and Engineering, University of Toronto, Toronto, Ontario, Canada.

[5]Interdisciplinary Center for Electron Microscopy, École Polytechnique Fédérale de Lausanne (EPFL), 1015 Lausanne, Switzerland.

[6]CLS@APS sector 20, Advanced Photon Source, Argonne National Laboratory, 9700 S. Cass Avenue, Argonne, IL 60439, USA.

[7]Canadian Light Source Inc., 44 Innovation Boulevard, Saskatoon, Saskatchewan S7N 2V3, Canada.

[8]Department of Chemistry, Simon Fraser University, Burnaby, British Columbia, Canada.

[9]These authors contributed equally: Erfan Shirzadi, Qiu Jin

*Corresponding authors. Email: ted.sargent@utoronto.ca, samira_siahrostami@sfu.ca.

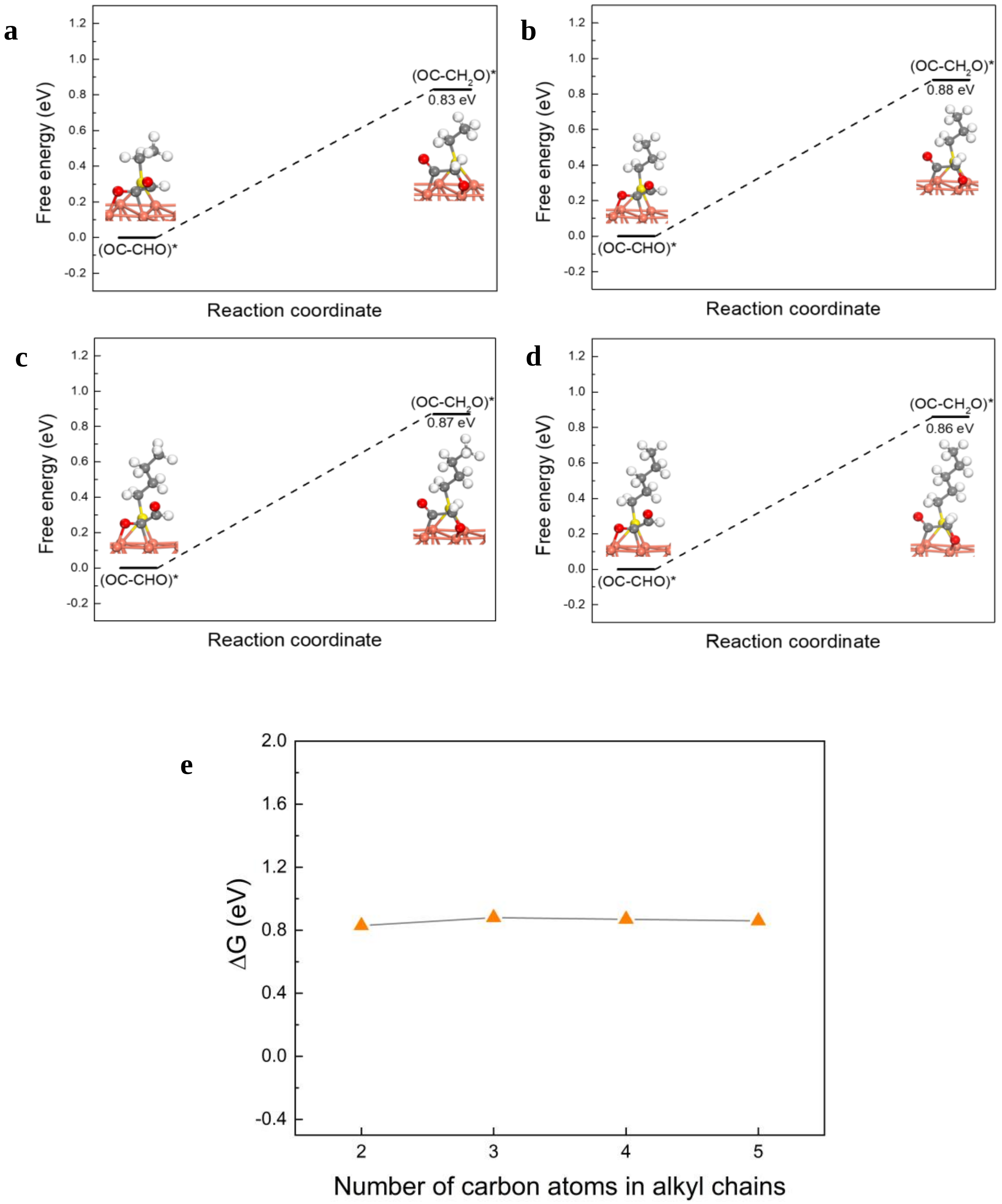

Supplementary Figure 1. Free energy diagram of the rate determining step from (OC-CHO)* to (OC-CH_2O)* over **a,** C_2S-Cu, **b,** C_3S-Cu, **c,** C_4S-Cu and **d,** C_5S-Cu slabs**. e,** Free energy change of the limiting step ((OC-CHO)* to (OC-CH_2O)*) as a function of the number of carbon atoms in alkyl chains attached to Cu surface. The comparable thermodynamic differences (maximum difference is 0.05 eV) indicates that the length of the alkyl chain has no effect on the earlier conclusions.

a

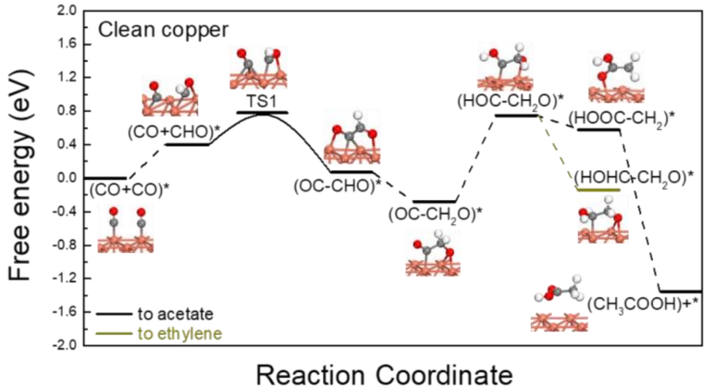

b

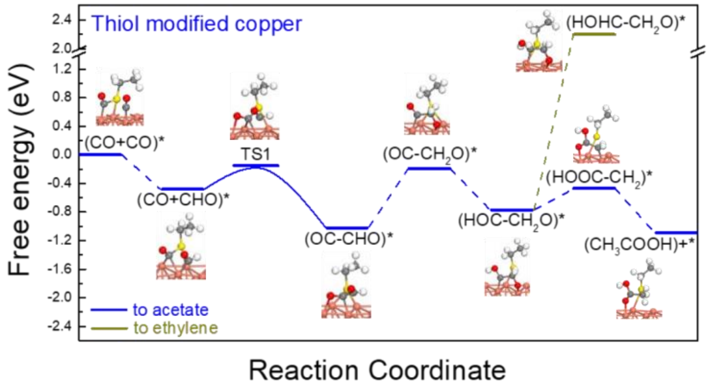

c

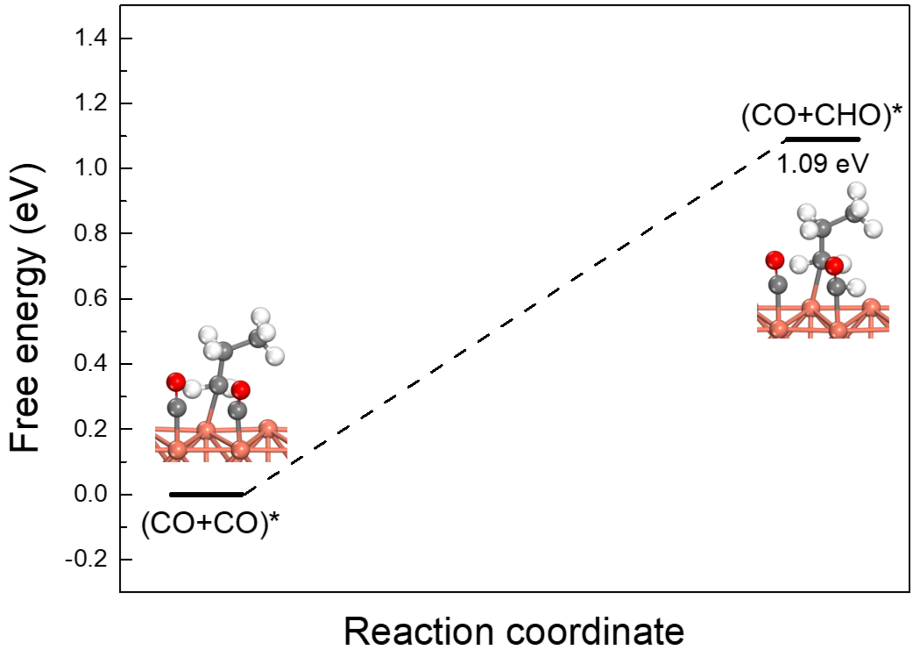

Supplementary Figure 2. Energy diagram of the conversion from CO to acetate over **a,** pure Cu and **b,** RS-Cu with 25 % thiol coverage. **c,**The hydrogenation of CO* adsorbate to CHO* over copper with propyl carbanion ligand attached.

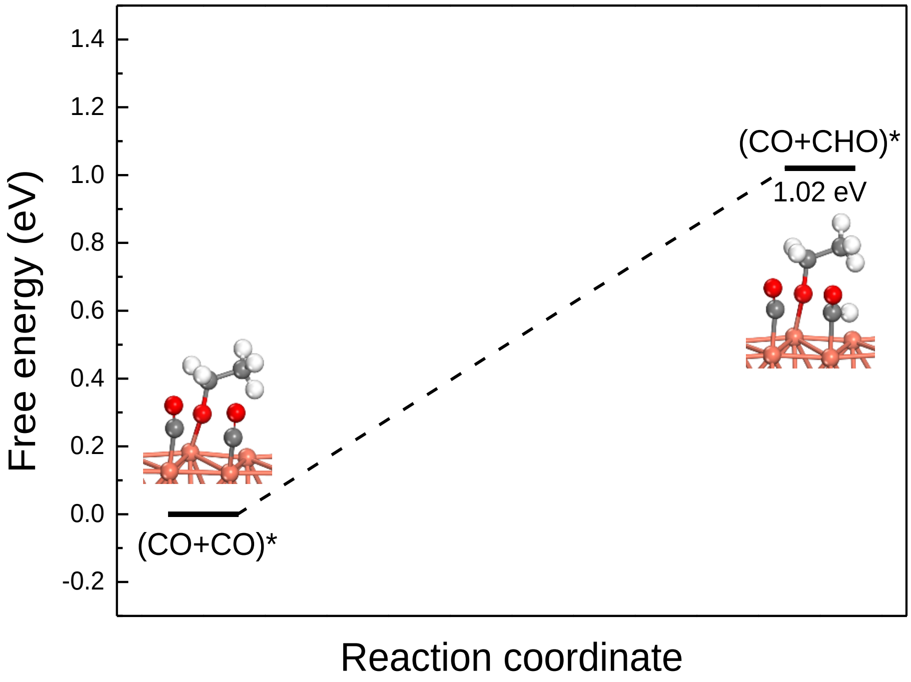

Supplementary Figure 3. The hydrogenation of CO* adsorbate to CHO* over copper with ethoxide ligand attached.

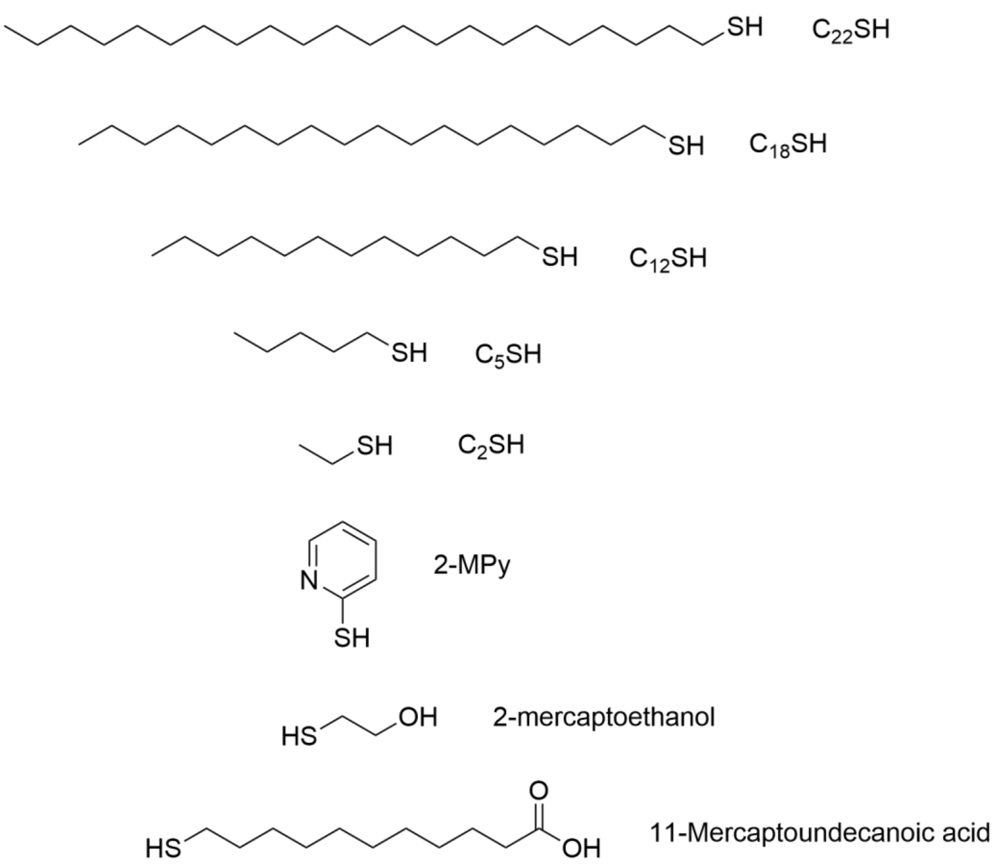

Supplementary Figure 4. Chemical structures of thiols that are studied in this work.

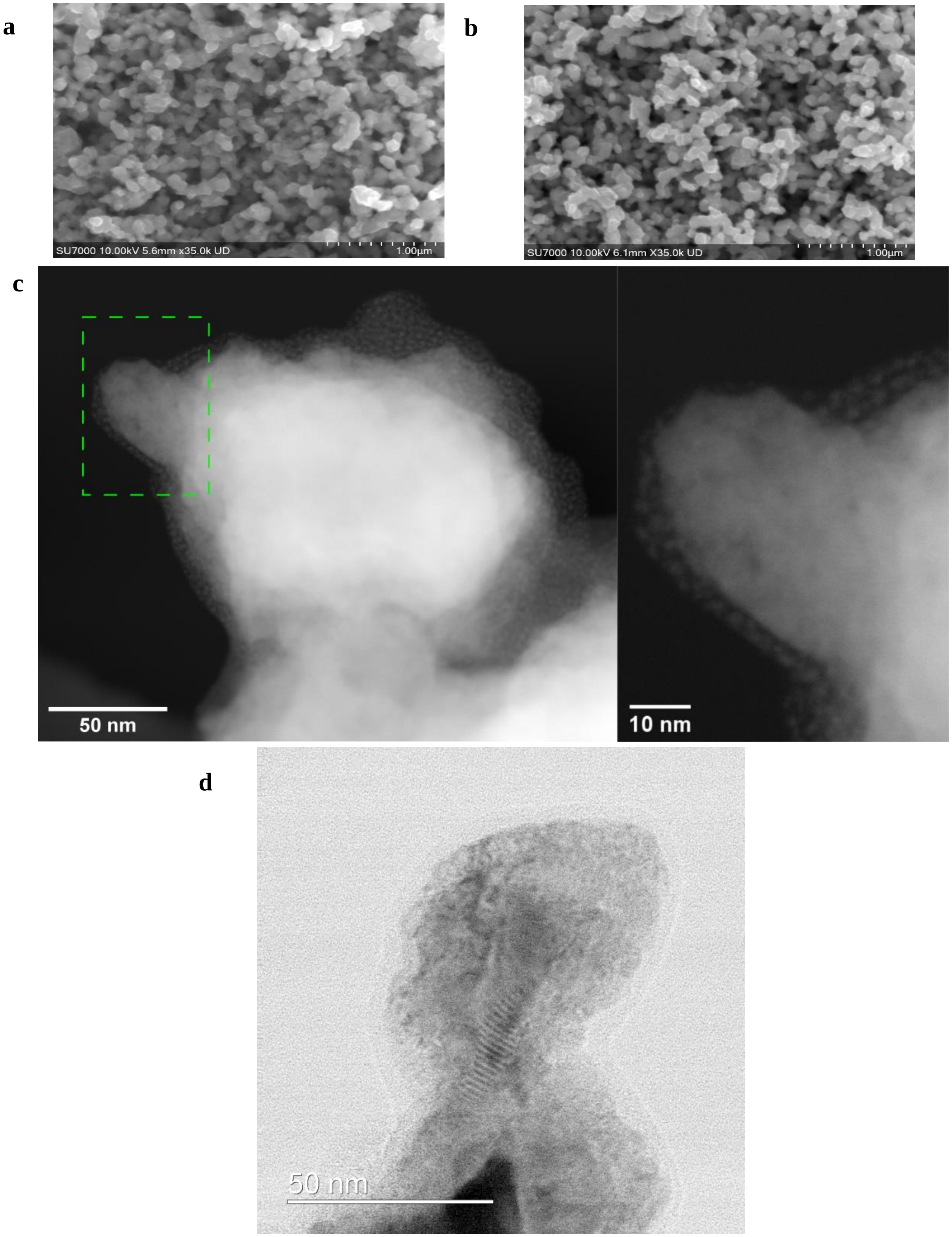

Supplementary Figure 5 SEM images of **a,** C_{18}S-Cu NPs and **b,** Cu NPs. **c,** Scanning transmission electron microscopy (STEM) high-angle annular dark-field (HAADF) image of C_{18}S-CuNPs.**d,** bright field STEM images of C_{18}S-CuNPs.

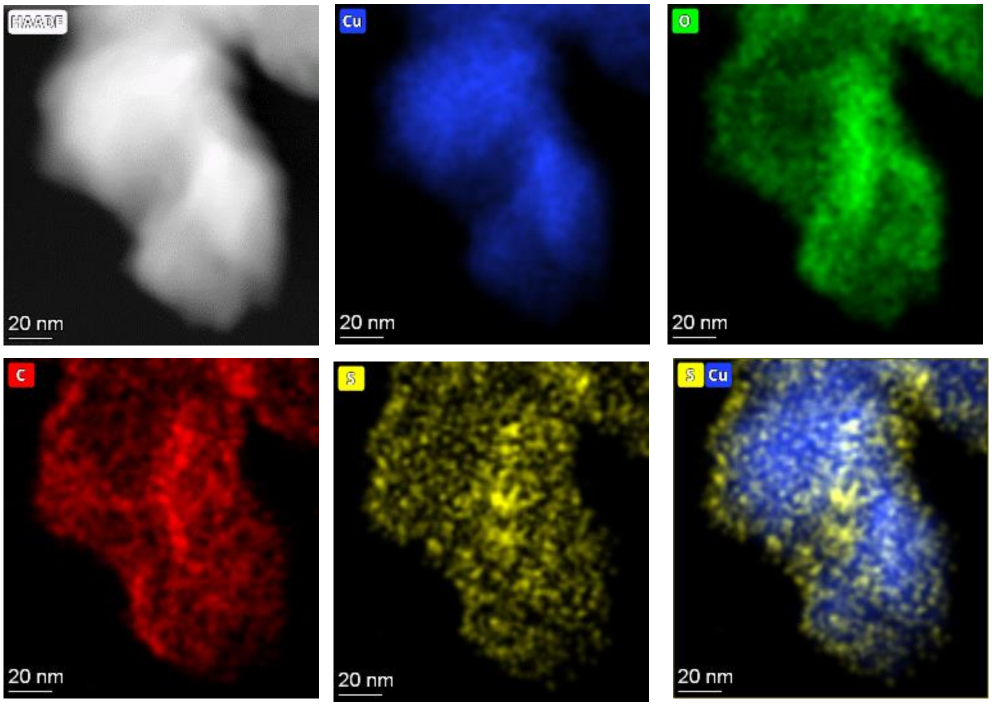

Supplementary Figure 6. STEM-EDX images of C_{18}S-Cu NPs which shows thiols as a shell layer around the nanoparticles.

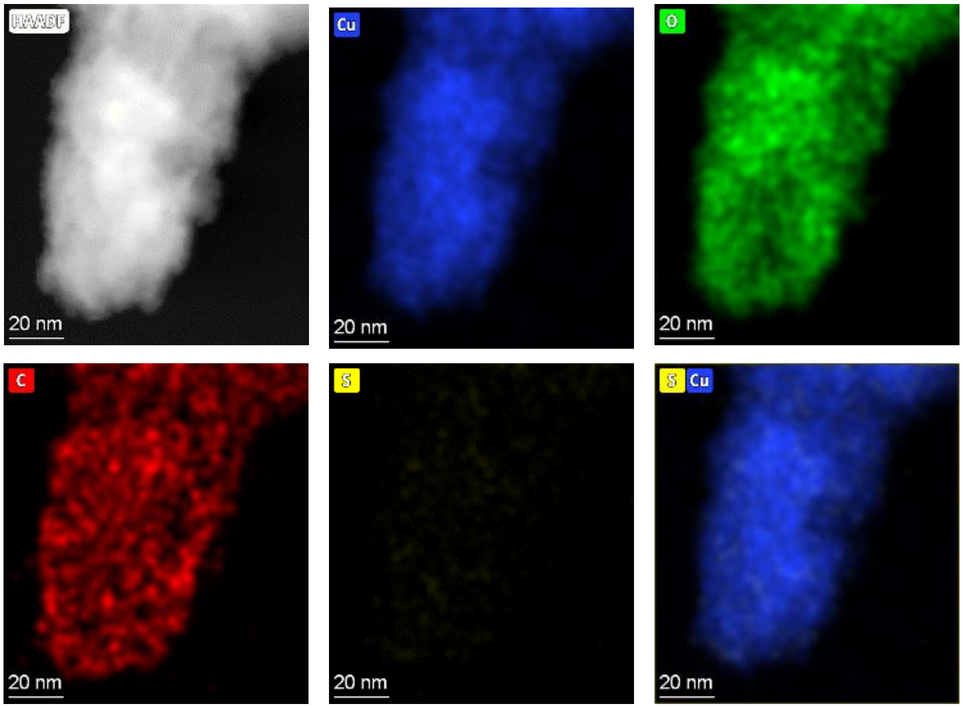

Supplementary Figure 7. STEM-EDX images of Cu NPs.

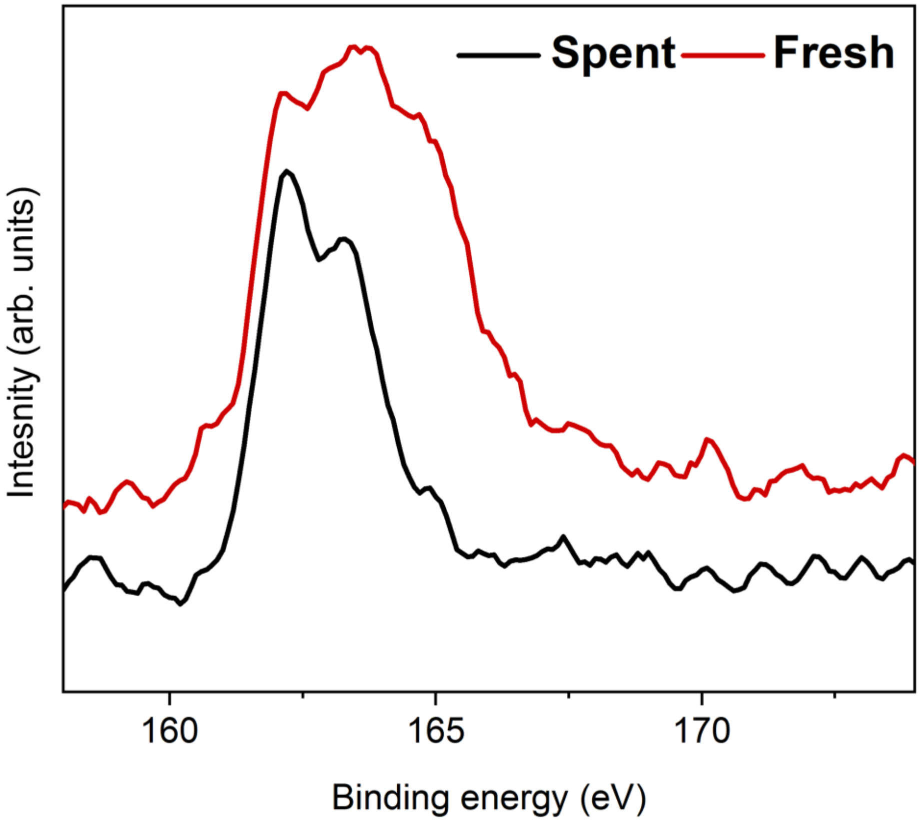

Supplementary Figure 8. Sulfur XPS spectra of C_{18}S-Cu NPs before and after CORR reaction showed that most chemisorbed thiols remained attached.

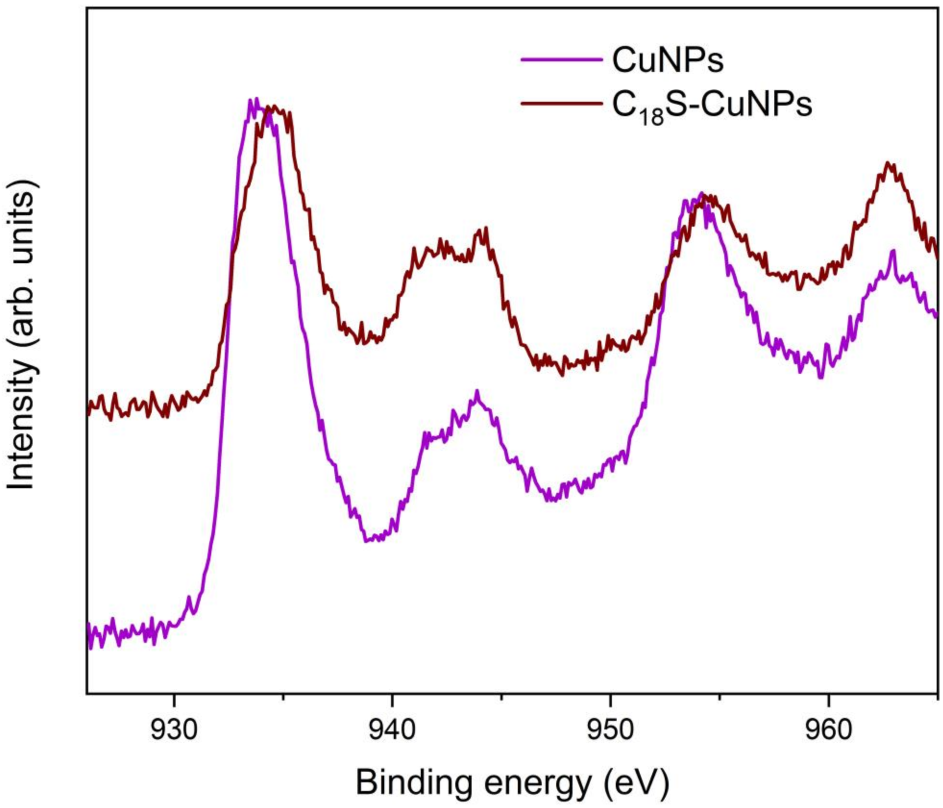

Supplementary Figure 9. Cu 2P XPS spectra of C_{18}S-Cu NPs and Cu NPs.

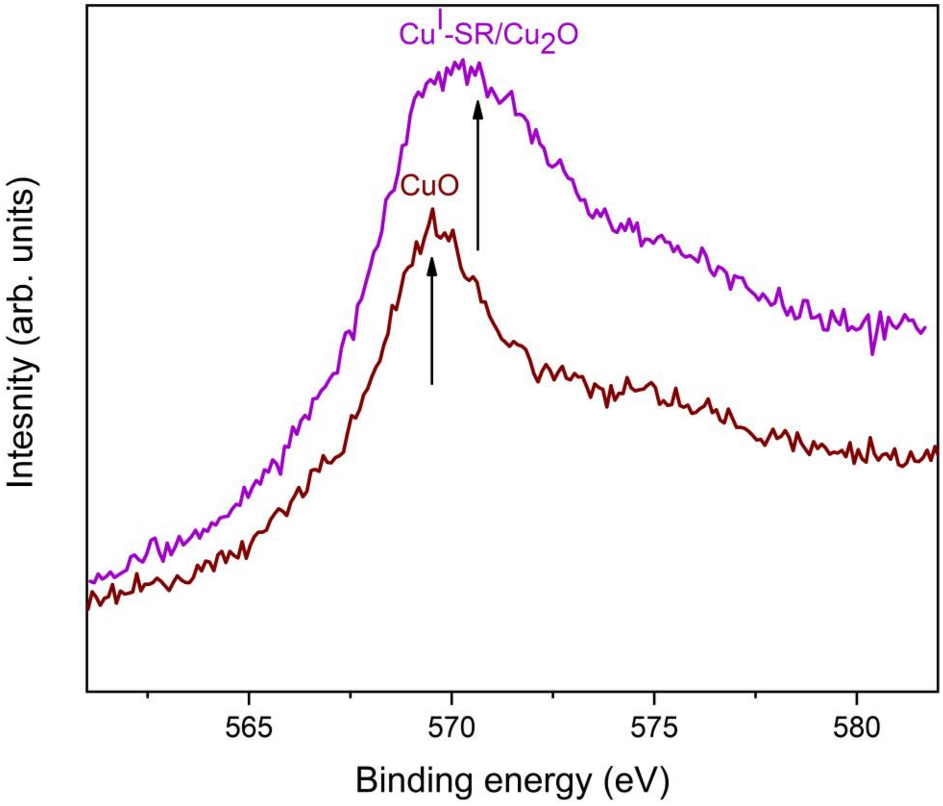

Supplementary Figure 10. CuLMM spectra of C_{18}S-Cu NPs and Cu NPs indicate a higher Cu^{I} in the C_{18}S-Cu NPs.

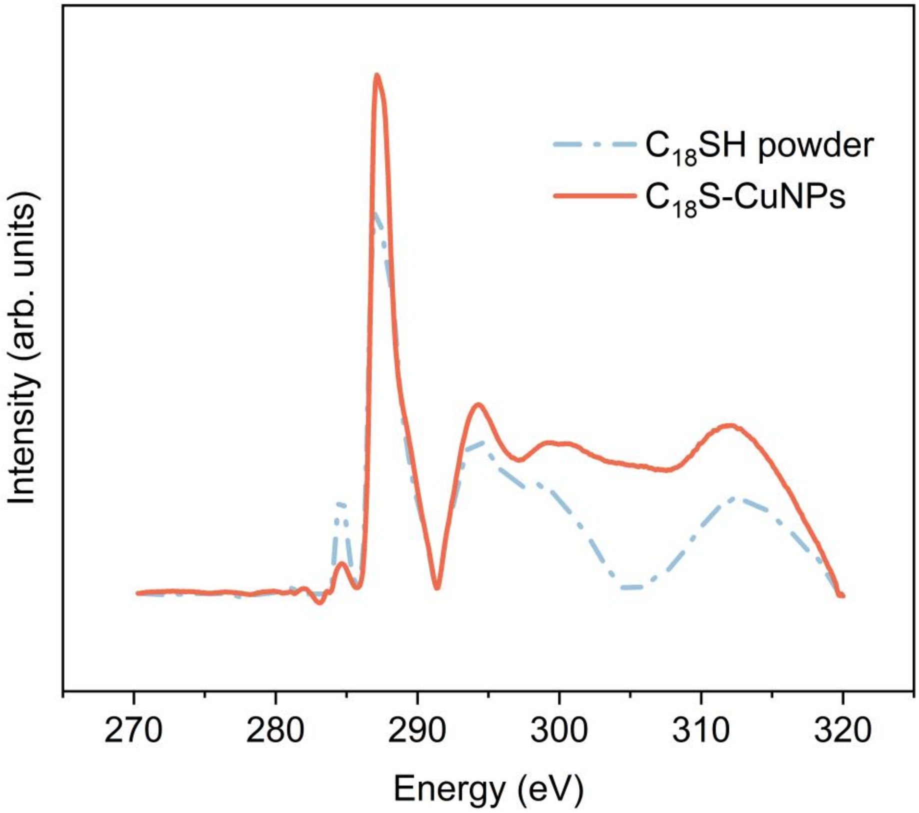

Supplementary Figure 11. Carbon K-edge TEY spectra of C_{18}S-Cu NPs and C_{18}SH powder show a higher orientation order of adsorbed C_{18}SH on copper than on C_{18}SH powder due to a sharper peak at 287 eV for C_{18}S-Cu NPs.

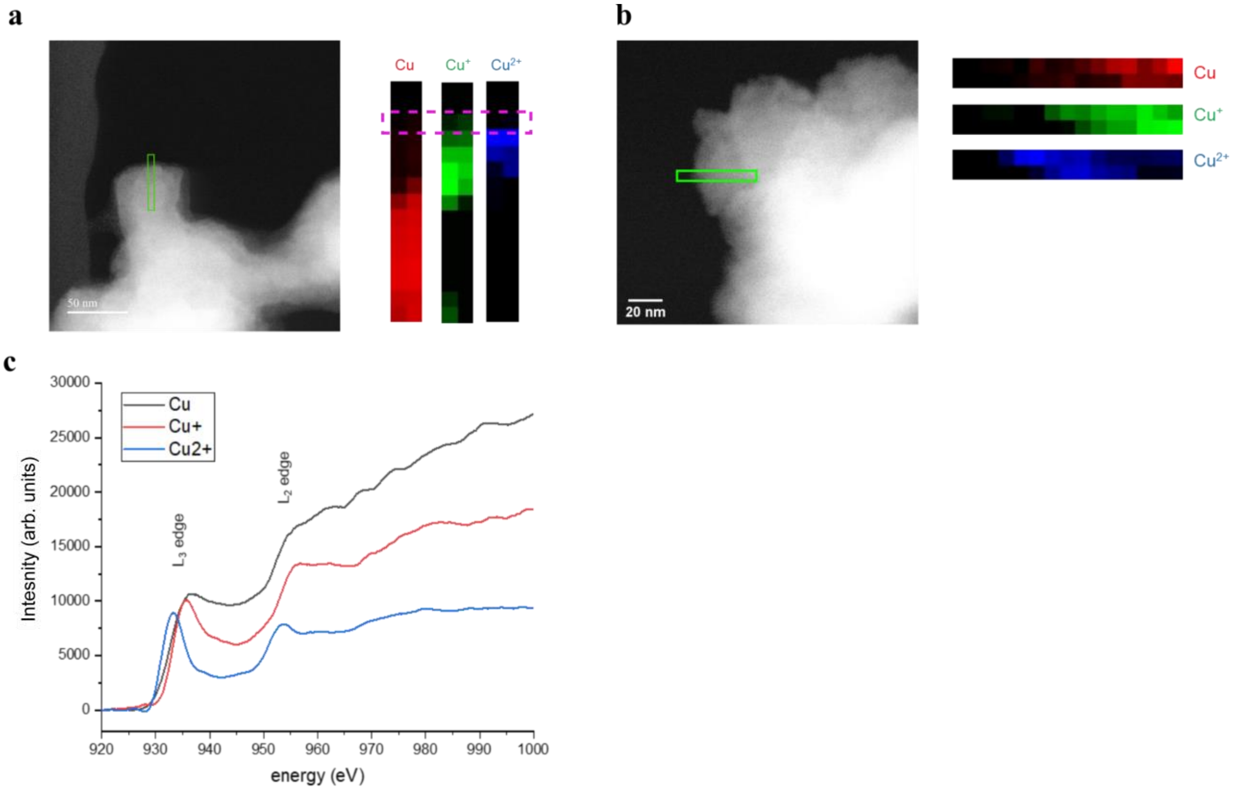

Supplementary Figure 12. a and b, Color maps of the Cu oxidation state, from EELS analysis of Cu L-edge, across particle surface as indicated on the STEM HAADF image. **a** is C_{18}S-Cu NPs show predominantly Cu^{I} whereas **b** is CuNPs show Cu^{II} oxidation state. This indicates that the surface of C_{18}S-Cu NPs contains Cu^{I}-S bonds. **c,** ELNES of the L edges of Cu, Cu^{+}, and Cu^{2+} were used as references for the MLLS fitting.

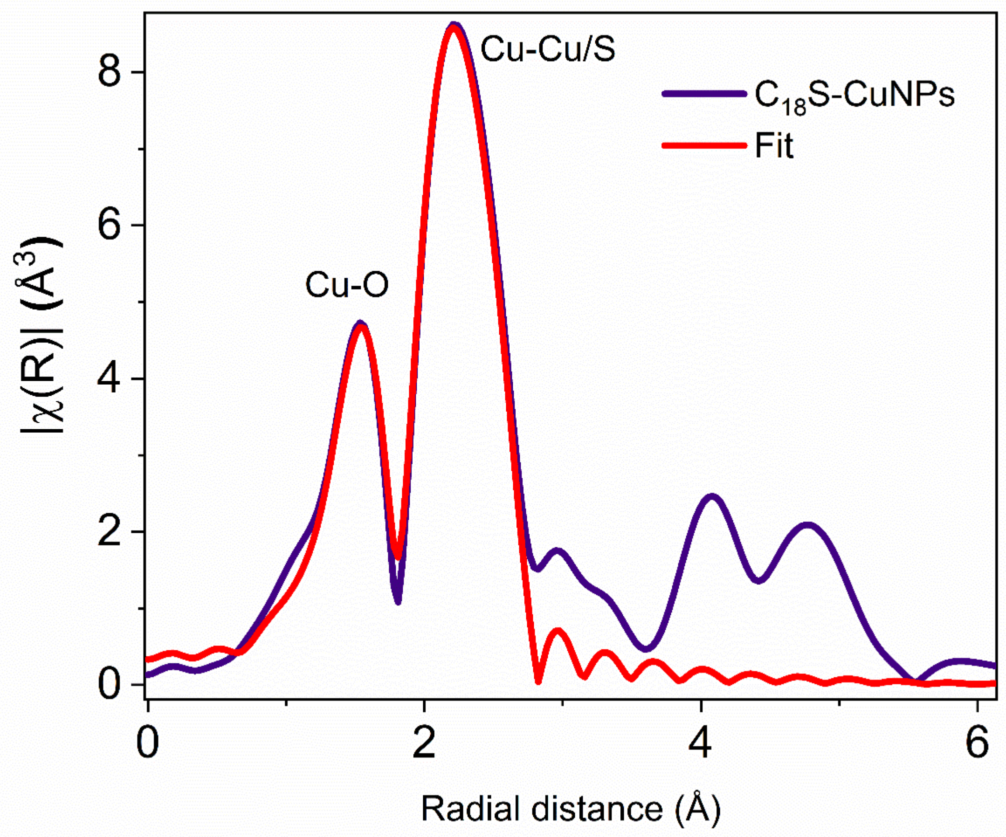

Supplementary Figure 13. *R*-space EXAFS spectra of $C_{18}S$-Cu NPs and fitted curve with slab presented in Supplementary Figure 11b.

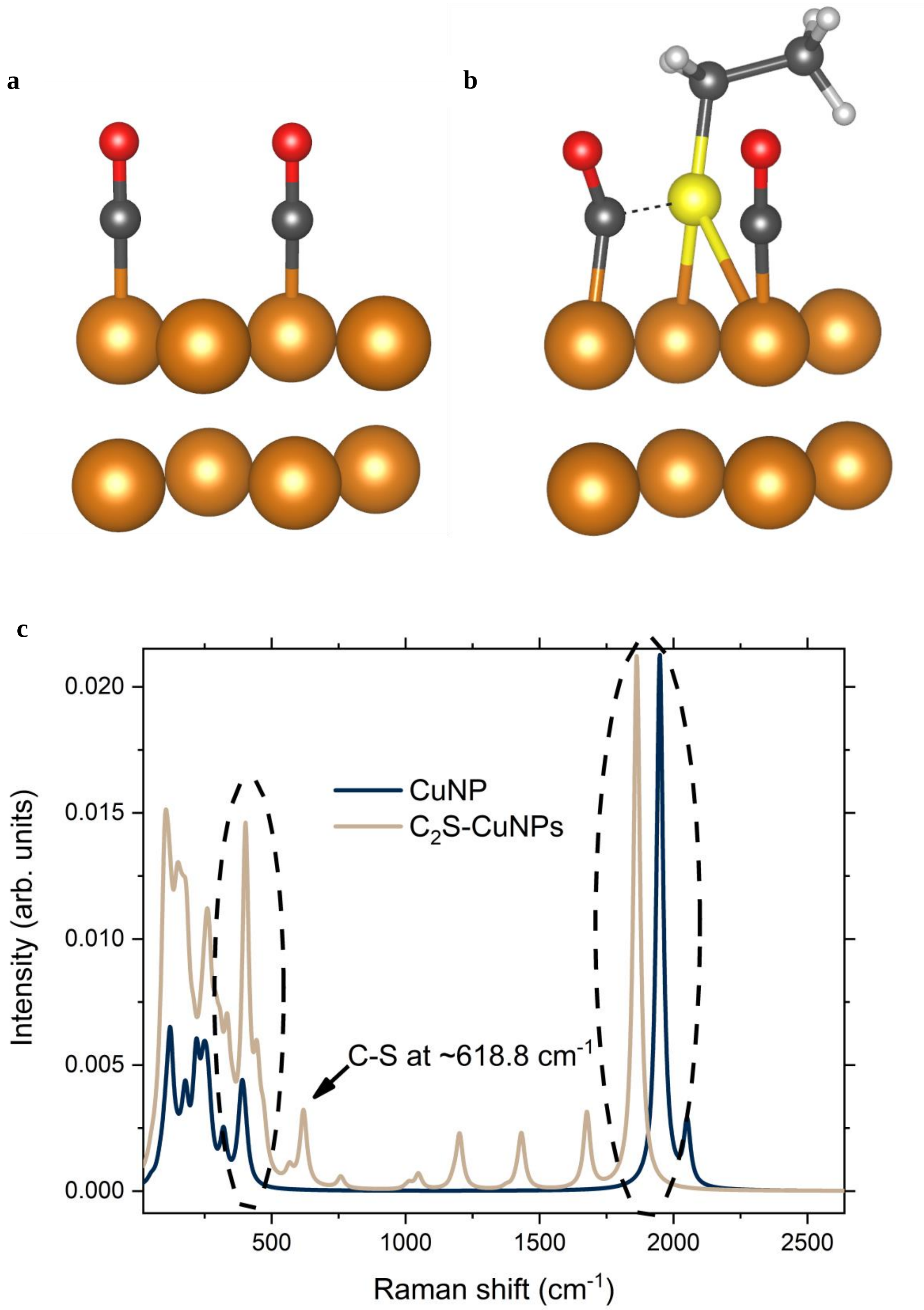

Supplementary Figure 14. Geometry-optimized CO on **a,** CuNP and **b,** C_2S-CuNPs slabs. **c,** Raman simulation results from the slabs shown in **a** and **b**.

a

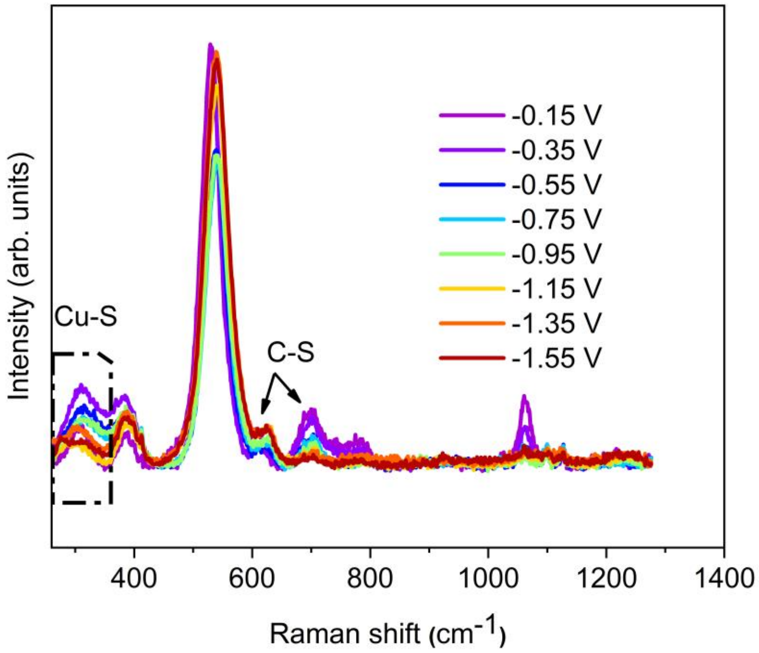

b

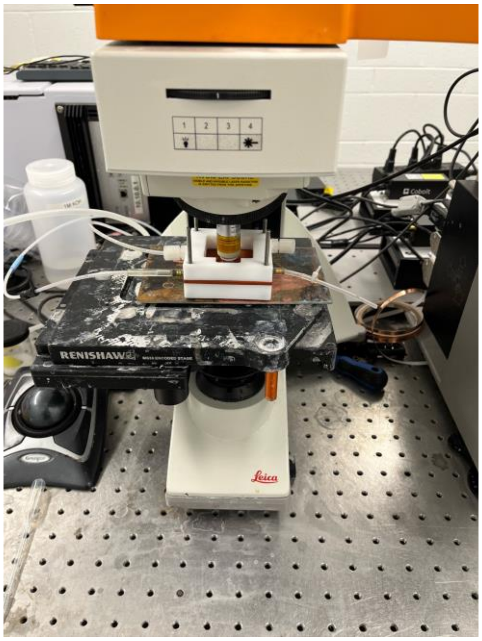

Supplementary Figure 15. **a,** In-situ Raman spectra of C_{18}SH-Cu NPs showing the presence of Cu-S bond up to -1.55 V vs. RHE. All the spectra are recorded in a CO atmosphere assembled in a flow cell and in contact with 5 M KOH without iR correction. **b,** The image of in-situ Raman spectroscopy setup.

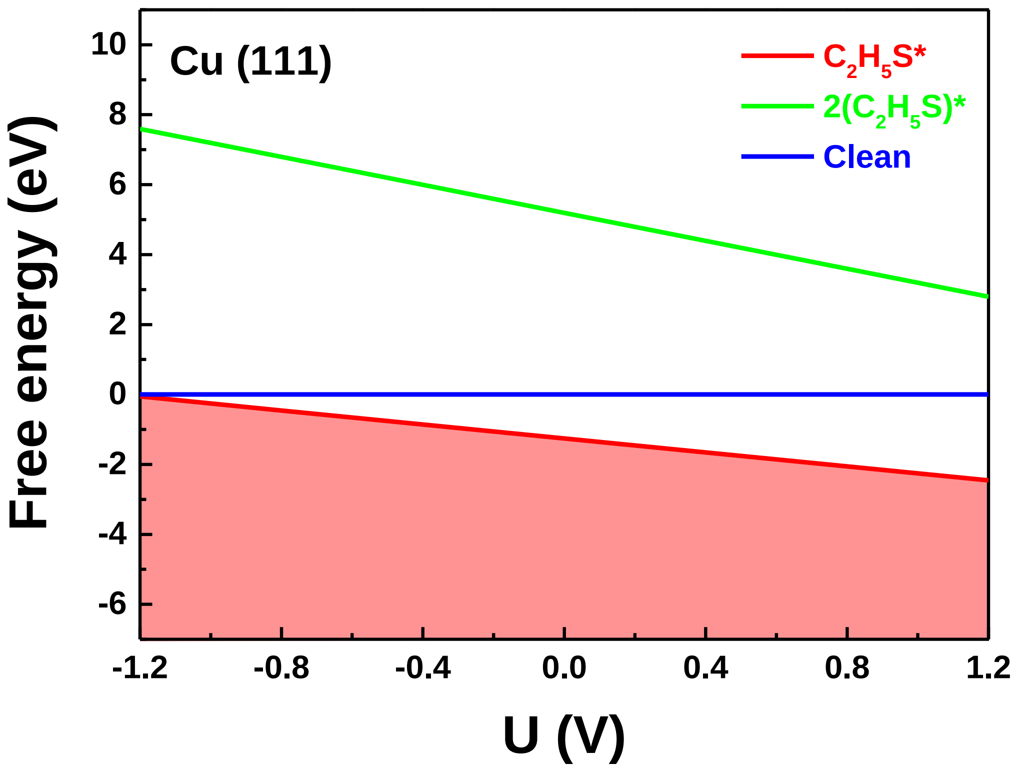

Supplementary Figure 16. Calculated surface free energy of Cu(111) as a function of electrode potential. The optimum number of the stably-adsorbed C_2H_5S on Cu(111) would be one under the potential range from -1.2 to 1.2 V.

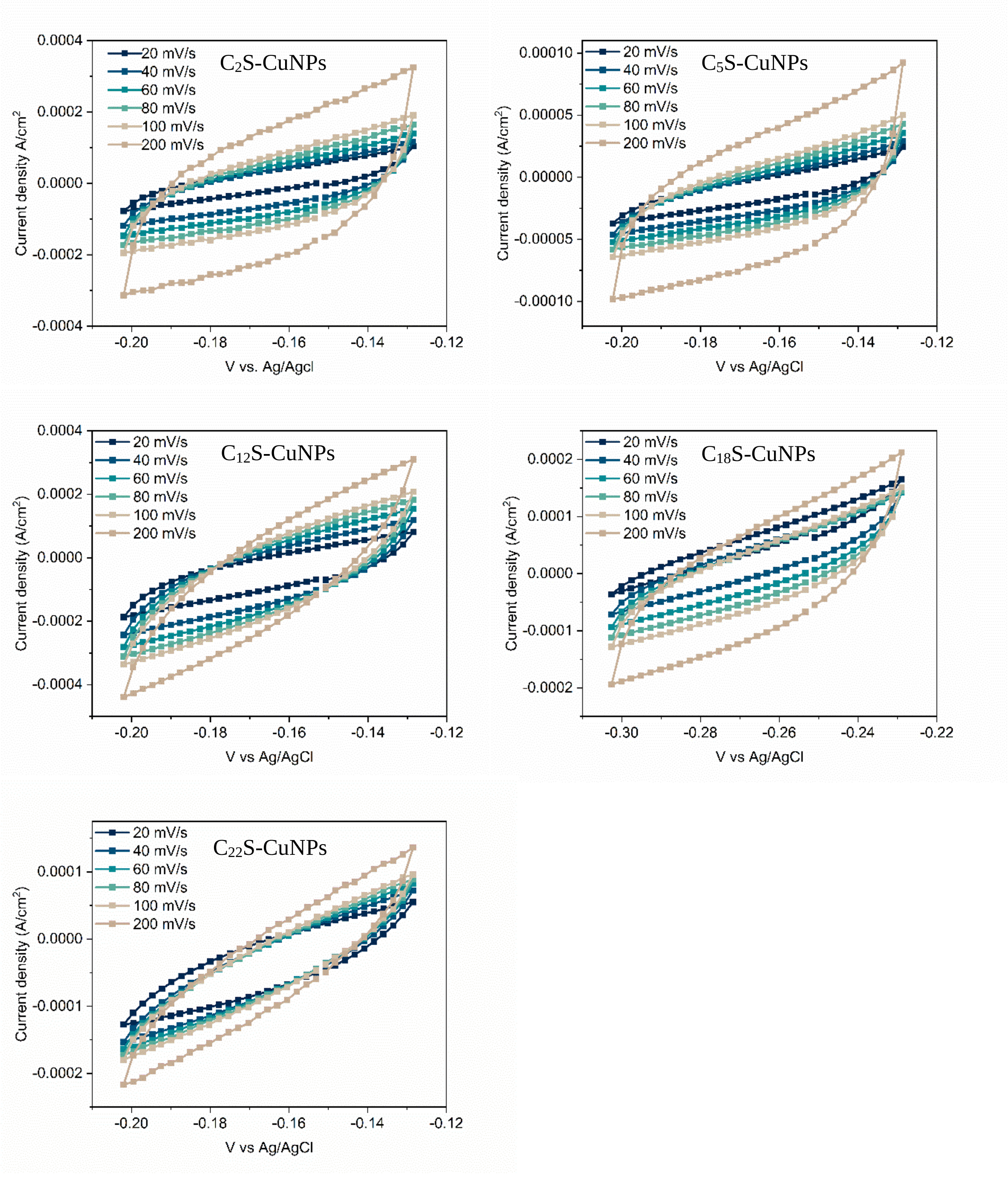

Supplementary Figure 17. Cyclic voltammetry curves of non-faradaic regions were obtained at different scan rates for RS-CuNPs. The voltages are not iR corrected.

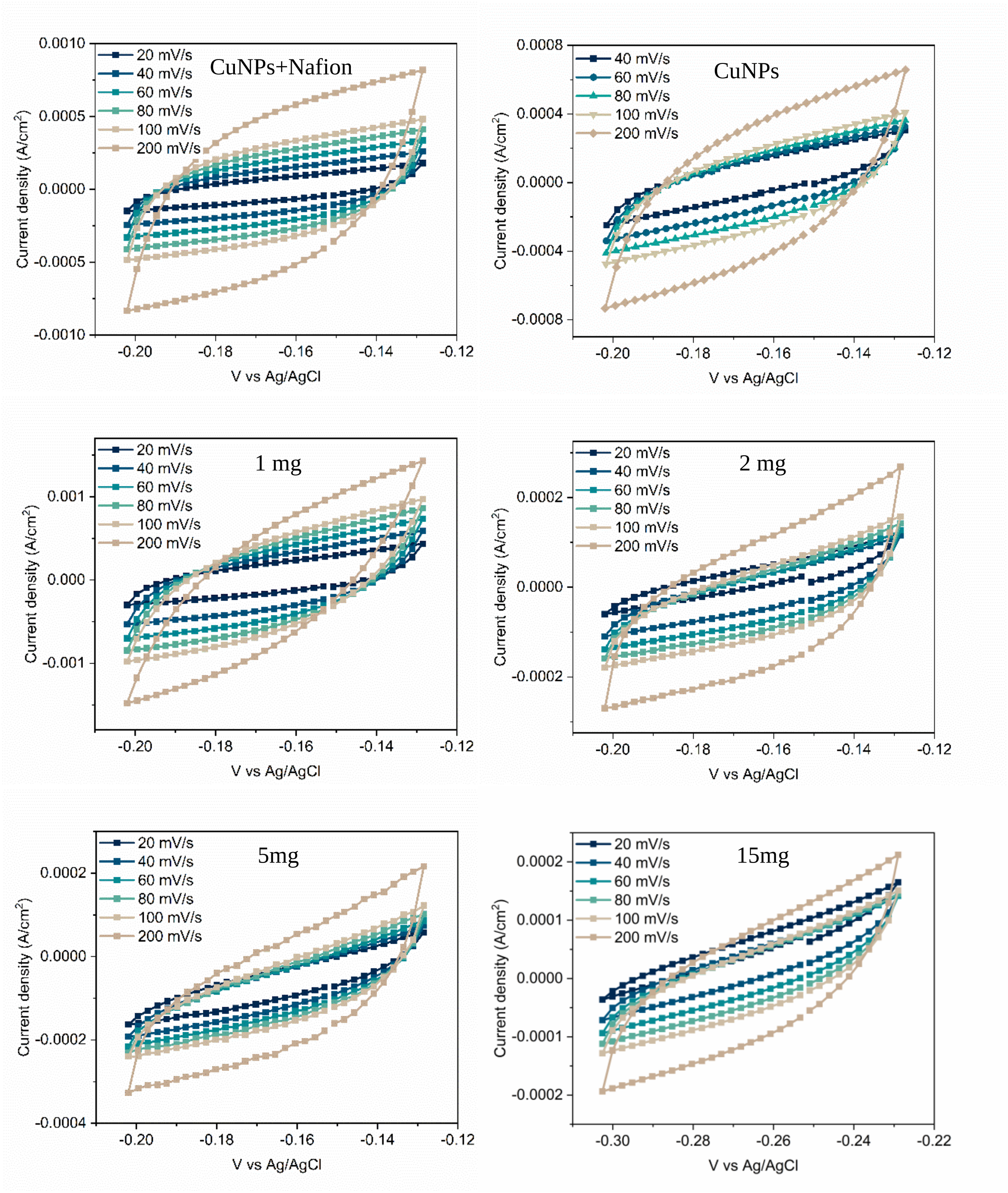

Supplementary Figure 18. Cyclic voltammetry curves of non-faradaic regions were obtained at different scan rates for Cu NPs and different loadings of C_{18}S-CuNPs.The voltages are not iR corrected.

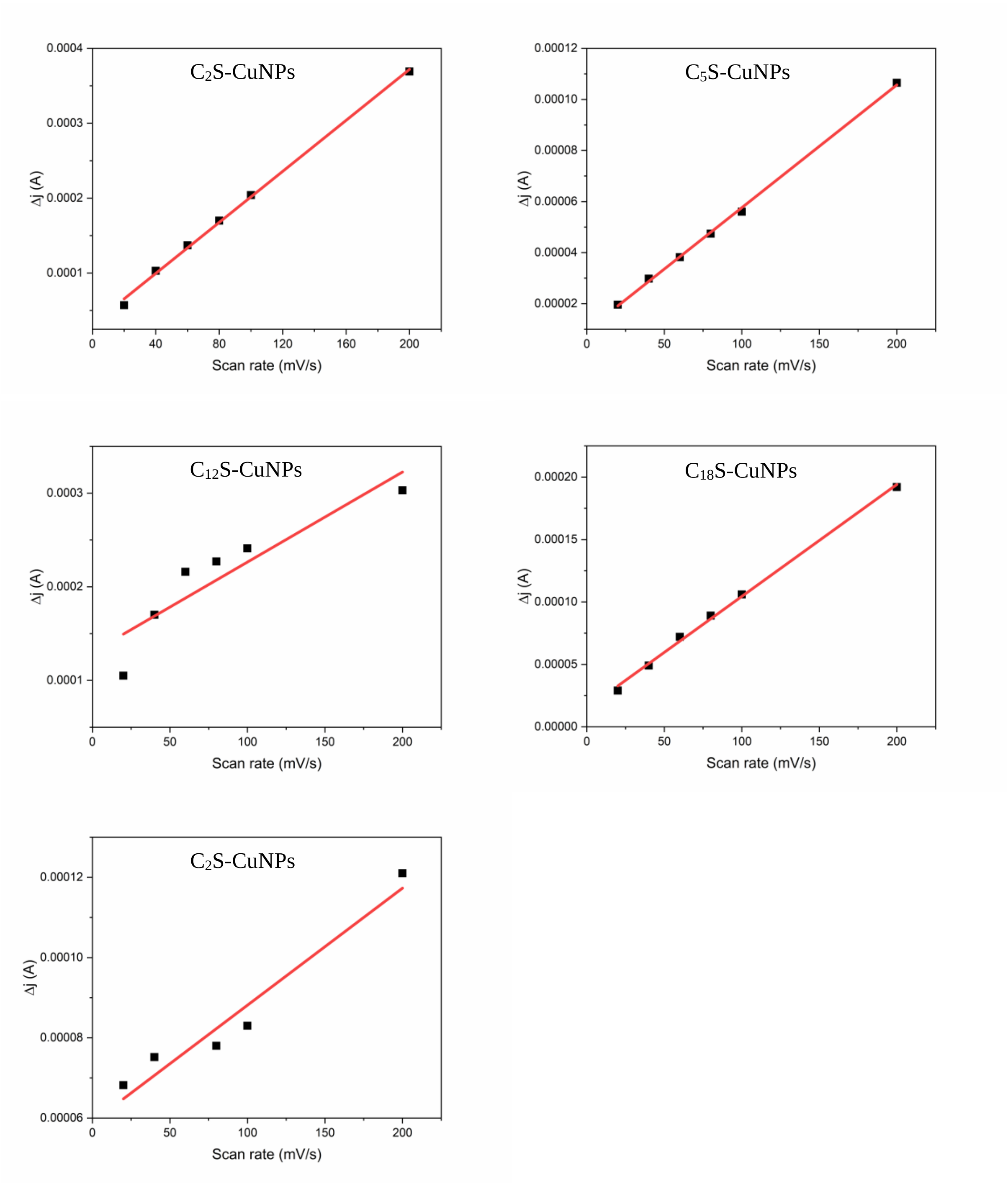

Supplementary Figure 19. Scan rate dependence of the current densities for RS-CuNPs.

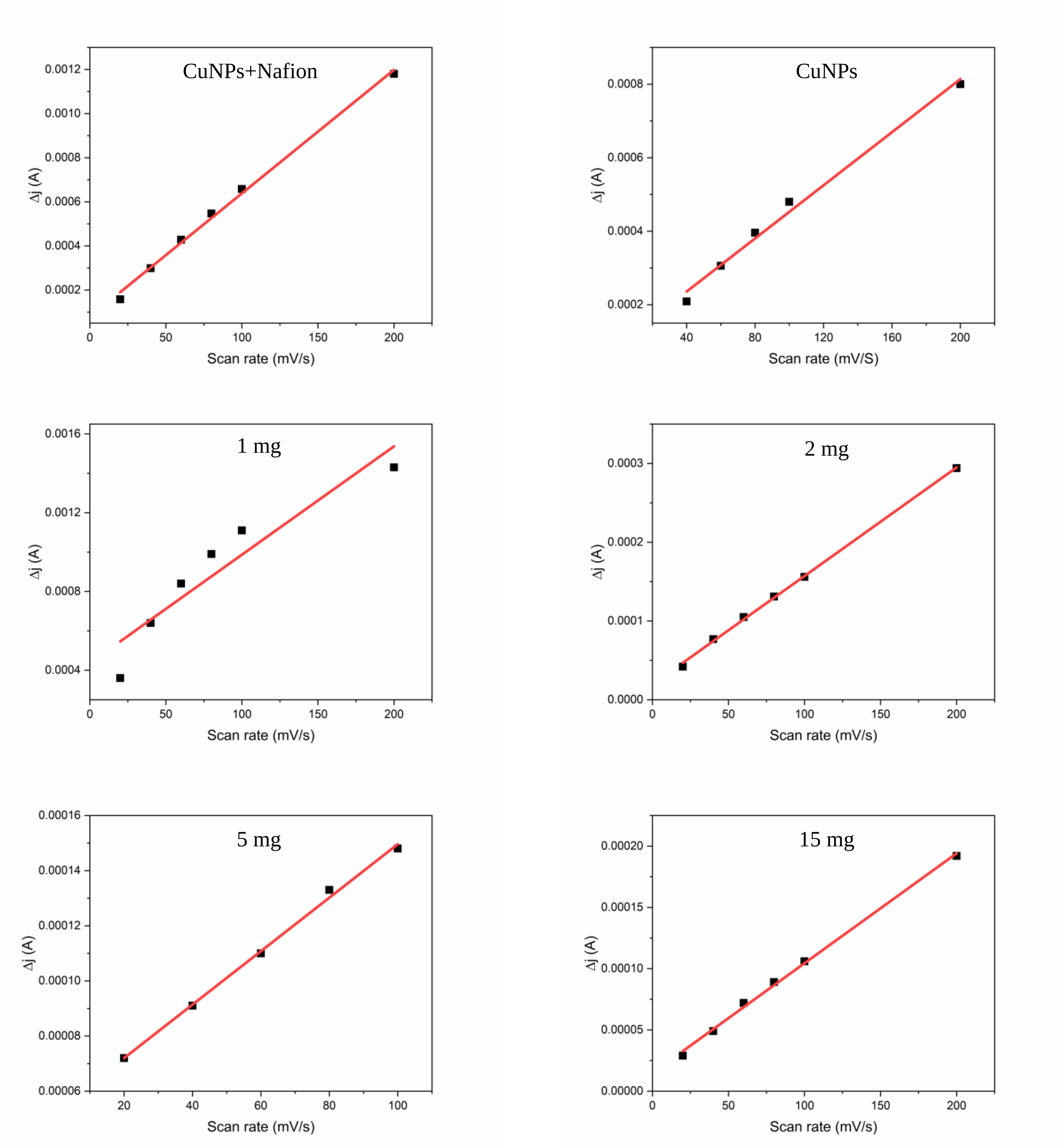

Supplementary Figure 20. Scan rate dependence of the current densities for Cu NPs and different loadings of C_{18}S-CuNPs.

a

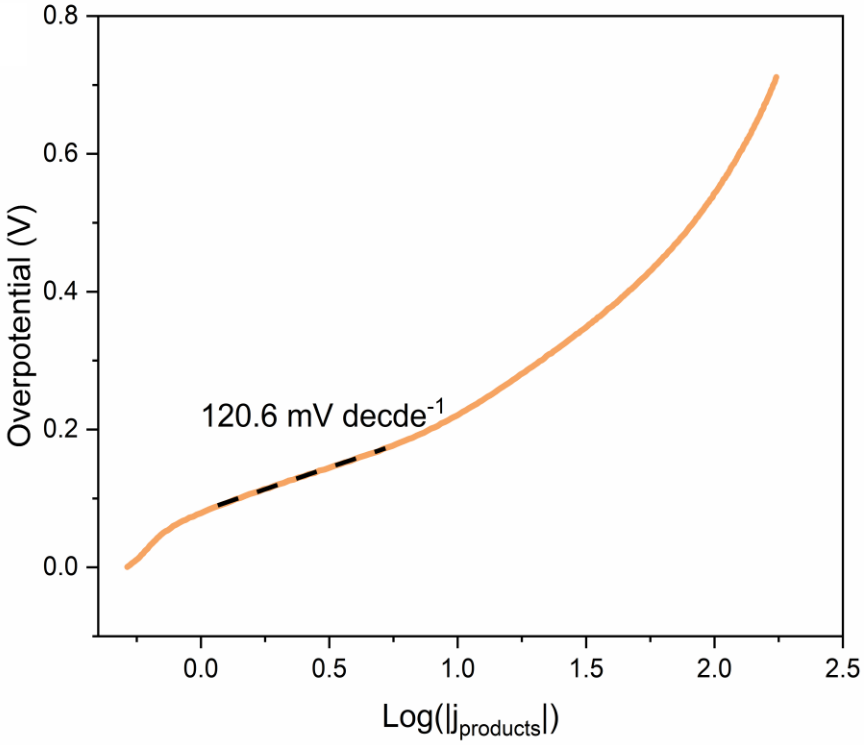

b

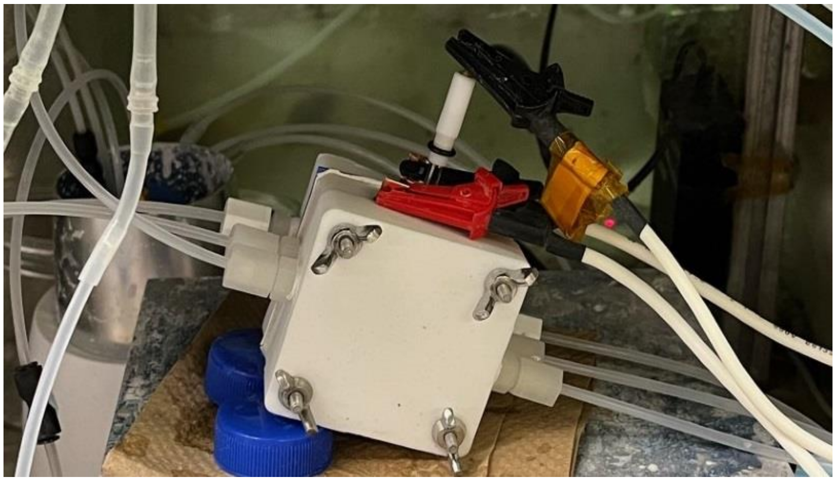

Supplementary Figure 21. **a,** Tafel slop analysis of C_{18}S-CuNP catalyst showing a Tafel slope of ~120mV.dec^{-1}, indicating the first electron transfer step to be the RDS. **b,** The flow-cell setup used for cyclic voltammetry and measurement of the electrochemical performance.

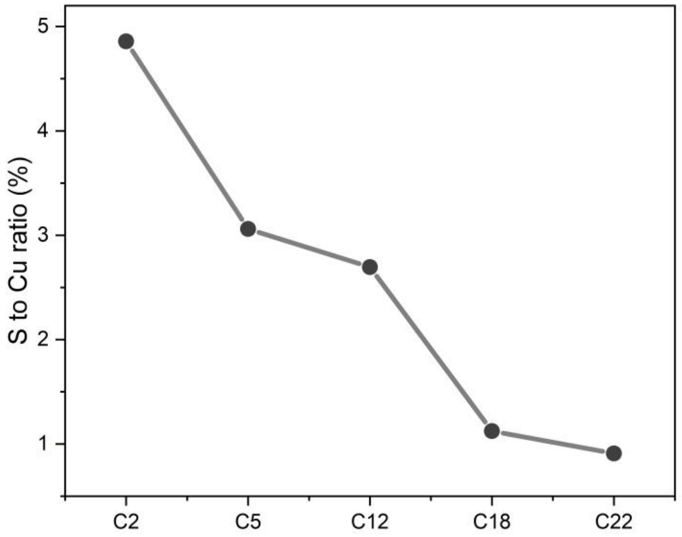

Supplementary Figure 22. The ICP-OES results indicate a reverse relation between the coverage and alkyl thiol length in RS-CuNPs catalysts.

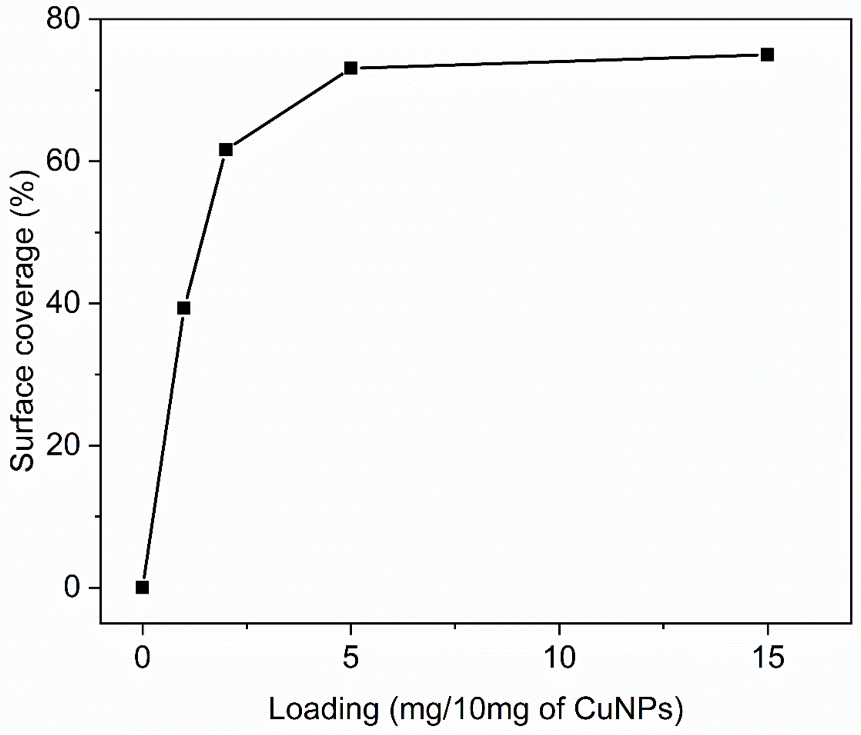

Supplementary Figure 23. The dependence between $C_{18}SH$ loading and estimated surface coverage based on ECSA. The surface coverage= $\frac{ECSA_{CuNP}-ECSA}{ECSA_{CuNP}}$. Note the relatively linear dependence up to 2 mg and saturation effect for 5 and 15 mg of loading.

a

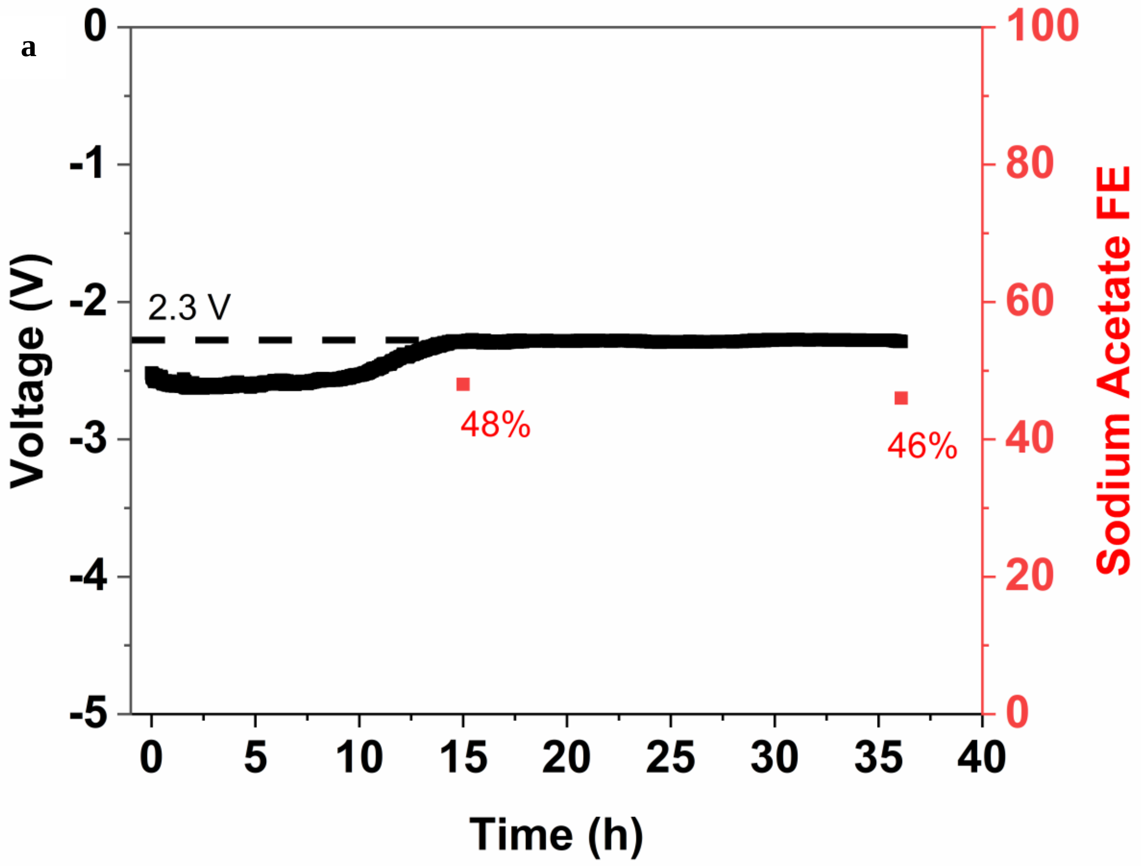

b

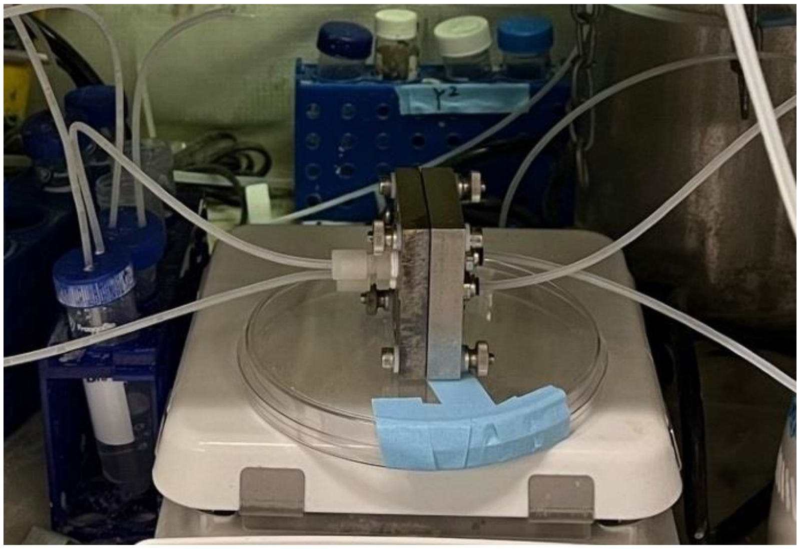

Supplementary Figure 24. a, Long-term operation test of C_{18}S-Cu NPs in an MEA system. An IrO_2 anode and a solution of 1M NaOH, and a Nafion cation exchange membrane were used. **b,** The image of the MEA setup used for the stability test.

Supplementary Note 1:

To further examine our hypothesis, we studied In-situ Raman spectroscopy on CuNPs treated with 2-mercaptopyridine (2-MPy). Due to the thione-thiol tautomerization and aromaticity, 2-MPy is not as good as $C_{18}SH$, and thus we expect a weaker bond between 2-MPY and copper. Supplementary Figure 25 shows the in-situ Raman spectra under a CO atmosphere. Based on a previous study on a similar thiol, the chemisorption on bulk copper happens at -0.2 V vs. RHE, which is near the potential of zero charge[1], a similar potential to what we observe the peak at ~310 cm^{-1} appears. We also noticed that as the *v(Cu-S)* peak grows, the peak related to *v(Cu-CO)* interacting with the thiol molecule appears at ~394cm^{-1}. This correlation supports our observations that the Cu-S bond interferes with the CORR catalysis.

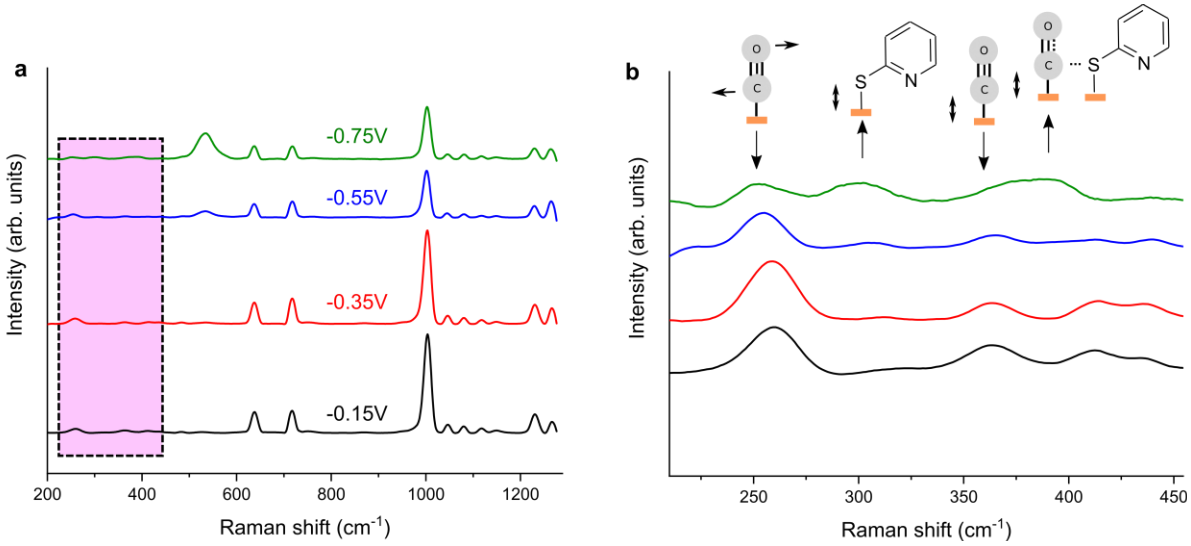

Supplementary Figure 25. a and b) In-situ Raman spectra of 2-MPy-CuNPs at various potentials versus RHE (not iR corrected) showing as the *v(Cu-S)* bond grows, a new peak related to *v(Cu-CO)* interaction with 2-MPy emerges at ~394 cm^{-1}.

Supplementary Note 2:

We performed in-situ XAS measurements on CuNPs and C_{18}S-CuNPs. Comparing C_{18}S-CuNP samples to CuNPs, including under reducing potentials, we observed a shift toward smaller atomic distances, a finding we assign to the contribution of the Cu-S bond in the EXAFS region (Supplementary Figure 26). A similar shift was observed in the EXAFS results of the sample after CORR (spent sample) (Supplementary Figure 27). We fit the EXAFS data of the spent sample with the simulation obtained from DFT-optimized C_2S-Cu slab (Supplementary Figure 14 and Supplementary Table 1) that suggests the origin of the shift observed for C_{18}S-CuNPs is the contribution of sulfur atom in the photo-electron scattering. In-situ X-ray absorption near-edge fine structure (XANES) of Cu K-edge revealed a shift to higher energy values for C_{18}S-CuNPs (see Supplementary Figure 28a). We explain this shift by considering the attachment of thiols causes a δ^+ charge on the adjacent copper atoms. Based on the DFT calculations, this charge can be as high as +0.11e (Supplementary Figure 29). To crosscheck with DFT studies, we estimated the average oxidation state of copper atoms under different conditions (Supplementary Figure 28b). We quantified the oxidation state of R-SCu NPs and CuNP under reduction conditions as +0.2 and 0 based on the linear relationship between copper K-edge and oxidation state[2].

We conducted XPS analysis of C_{18}S-CuNP samples that had undergone CORR. We noticed a narrower peak in S*2p* region confirming the removal of physisorbed alkanethiols during the electrochemical reaction (Supplementary Figure 8). This is due to the reaction of RSH as a weak acid with the alkaline electrolyte used for CORR. Based on XPS analysis, we observed a 1 to 4 ratio between S and Cu, a coverage similar to what we used in our DFT calculations (Supplementary Table 2).

a

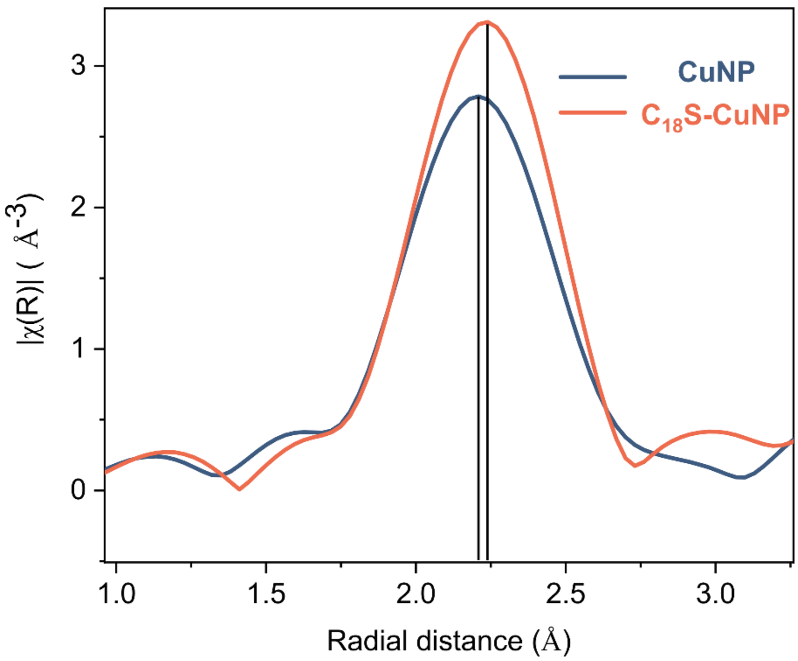

b

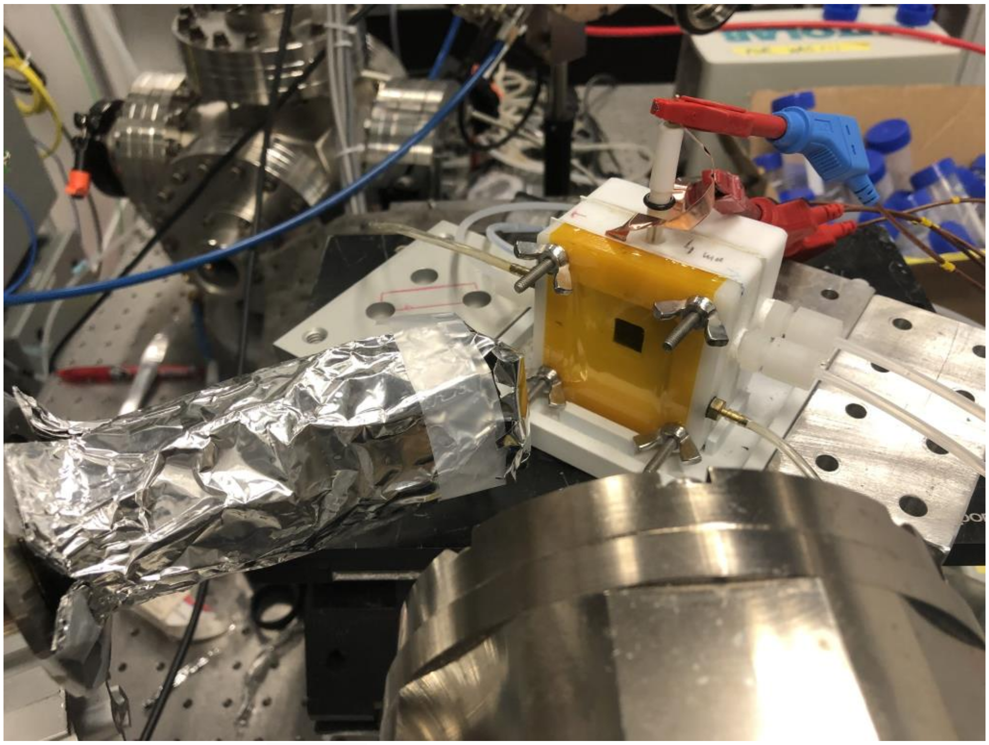

Supplementary Figure 26. **a,** *R*-space EXAFS spectra of C_{18}S-Cu NPs and Cu NPs under reduction conditions measured at -1.7 V Vs. Ag/AgCl (not iR corrected). **b,** The image of the in-situ XAS setup.

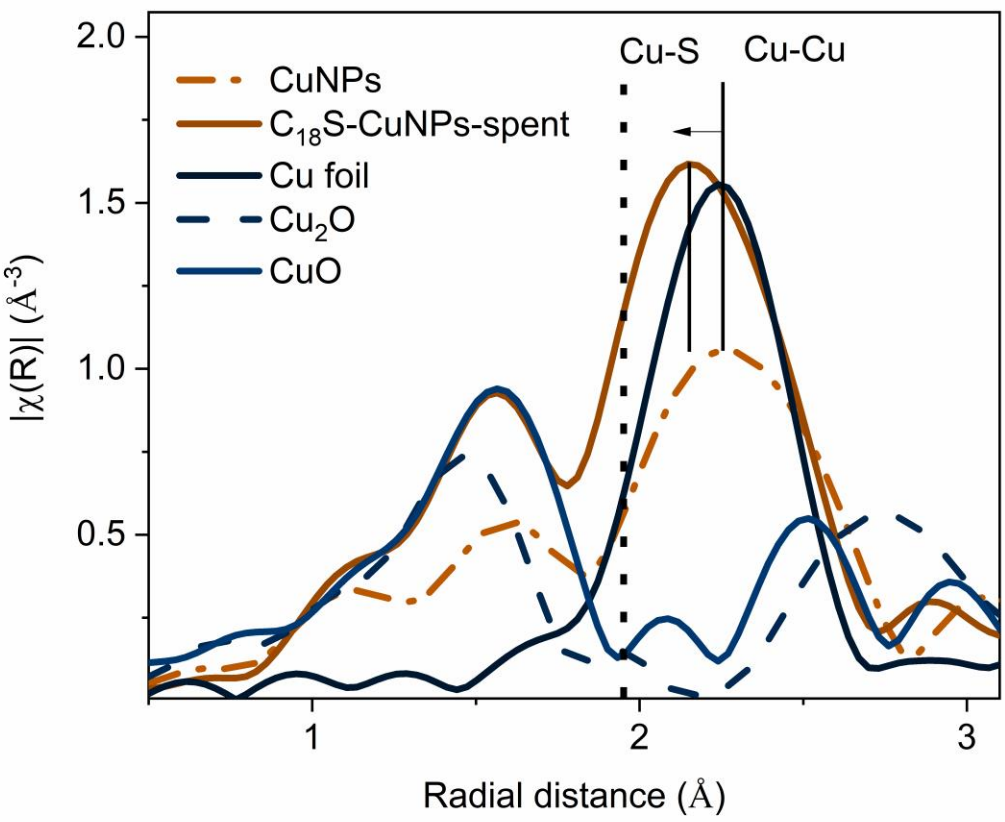

Supplementary Figure 27. *R*-space EXAFS spectra of spent-C_{18}S-Cu NPs and Cu NPs.

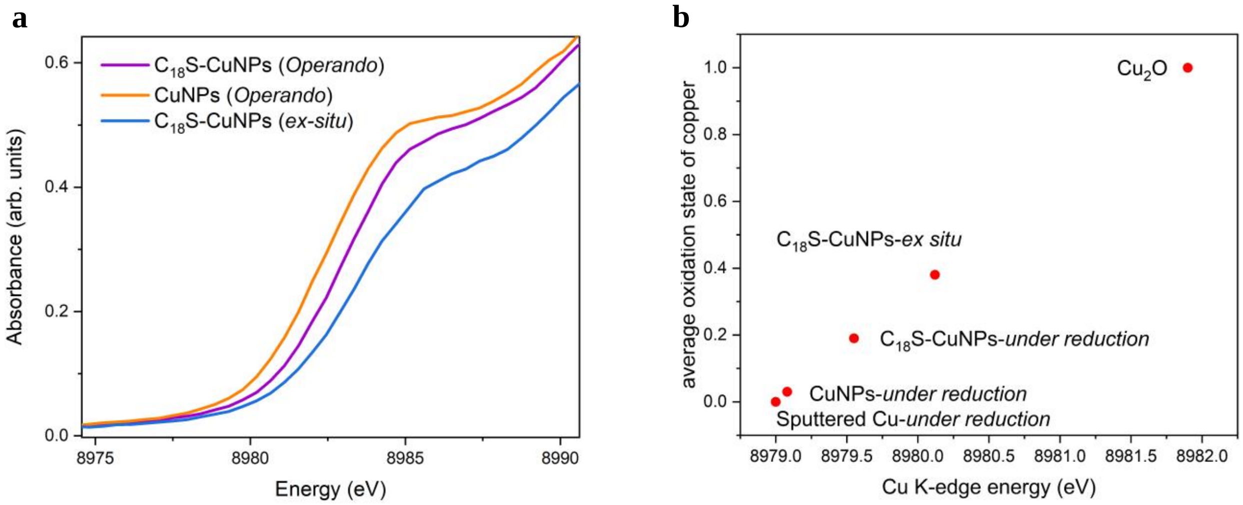

Supplementary Figure 28. a) XANES spectra of C_{18}S-Cu NPs in operando and ex-situ conditions compared with Cu NPs measured at -1.7 V Vs. Ag/AgCl (not iR corrected). b) The estimated average oxidation state of catalysts under *in situ* and *ex situ* conditions. The position of Cu_2O K-edge is based on the data previously published[3].

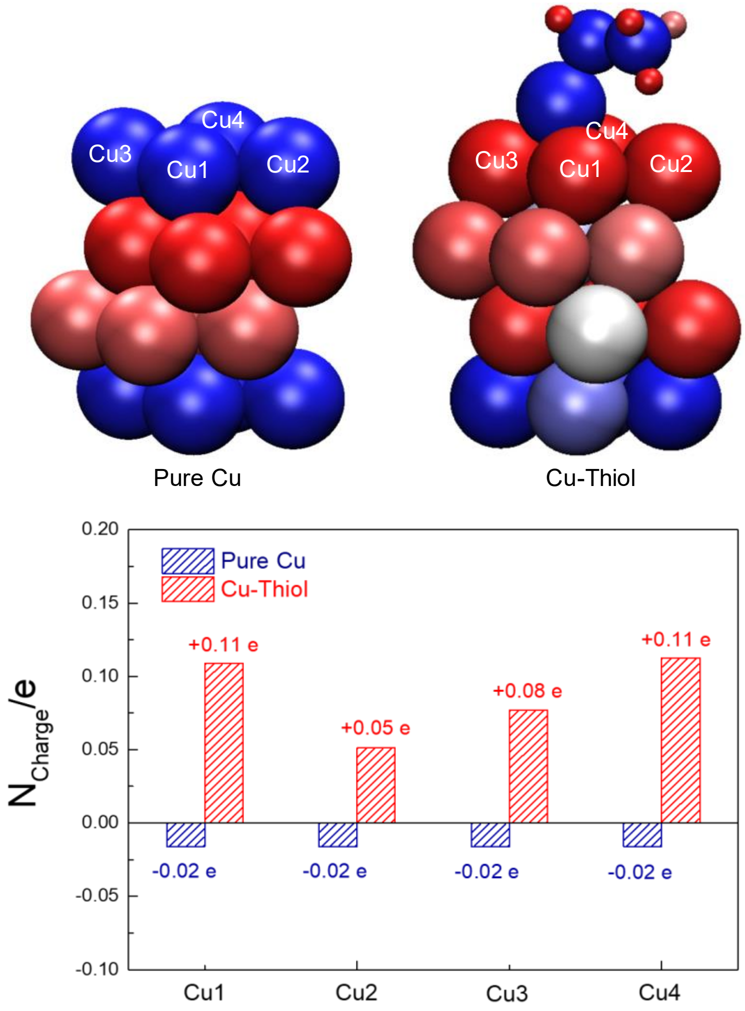

Supplementary Figure 29. Bader charge analysis of surface Cu atoms in pure Cu and thiolated Cu.

Supplementary Table 1. Resulting fitting parameters for EXAFS region of $C_{18}S$-CuNPs.

Fit parameter	Value
E_0	8.14 +/-1.7
ΔR	0.0107 +/- 0.0110
σ	0.00657 +/- 0.00069

Supplementary Table 2. Elemental composition of spent $C_{18}S$-CuNP sample based on XPS data.

**Element**	**Atomic%**
**C**	58.4%
**O**	31%
**Cu**	8.5%
**S**	2.2%

Supplementary Table 3. Summary of Faradaic efficiencies of different surface ligand-based CuNP catalysts at 100mA/cm^2.

Catalyst	H_2 FE (%)	C_2H_4 FE (%)	CH_4 FE (%)	C_2H_5OH FE (%)	C_3H_7OH FE (%)	CH_3COO^- FE (%)
**C_2S-Cu NPs**	27.3±4.2	22.9±0.9	0.0±0.0	16.9±2.0	3.0±0.7	24.1±2.2
**C_5S-Cu NPs**	7.1±1.2	24.3±1.3	0.9±0.2	10.2±1.4	3.3±1.3	40.8±3.6
**C_{12}S-Cu NPs**	7.6±1.6	18.7±2.0	0.0±0.0	12.6±1.7	12.2±1.9	45.4±3.7
**C_{18}S-Cu NPs**	5.6±1.5	19.2±2.5	0.0±0.0	13.4±1.3	8.2±0.9	46.1±3.2
**C_{22}S-Cu NPs**	5.0±1.2	26.5±2.8	0.0±0.0	13.0±2.2	4.3±1.1	37.7±3.0
**Cu NPs**	39.7±2.3	10.8±1.2	0.0±0.0	7.4±1.8	5.4±0.8	13.5±1.9
**Cu NPs+Nafion**	9.4±1.1	37.8±2.3	0.0±0.0	13.6±1.5	15.3±2.1	14.4±1.3
**2-MPy-Cu NPs**	25.7±3.2	24.5±2.7	0.4±0.3	8.5±2.1	1.0±0.5	28.8±3.0
**2-Mercaptoethanol**	26.8±2.9	20.4±2.2	0.0±0.0	9.1±1.6	2.3±0.9	25.8±4.1
**11-Mercaptoundecanoic acid**	20.6±1.8	21.7±1.9	0.0±0.0	2.5±0.9	13.2±1.2	23.2±1.5

Supplementary Table 4. The ECSA, contact angle, and operating voltage of the RS-CuNPs.

Catalyst	Advancing Contact angle (degrees)	Receding Contact angle (degrees)	ECSA (cm^2_{ECSA})	Voltage vs Ag/AgCl at 100 mA/cm^2 (not IR corrected)
**C_2S-Cu NPs**	163.4	0	29.3	-1.63
**C_5S-Cu NPs**	170.3	163.7	8.3	-1.64
**C_{12}S-Cu NPs**	166.7	162.8	16.5	-1.66
**C_{18}S-Cu NPs**	169	163	15.5	-1.67
**C_{22}S-Cu NPs**	169.6	161	5.0	-1.74
**Cu NPs**	hydrophile	hydrophile	62.0	-1.80
**Cu NPs+Nafion**	-	-	96.6	-1.75

Supplementary Table 5. Summary of Faradaic efficiencies of RS-CuNP catalysts at 200mA/cm^2.

**Catalyst**	**H_2 FE (%)**	**C_2H_4 FE (%)**	**CH_4 FE (%)**	**C_2H_5OH FE (%)**	**C_3H_7OH FE (%)**	**CH_3COO^- FE (%)**
**C_2S-Cu NPs**	60.0±4.7	6.2±0.9	0.7±0.3	2.3±1.1	0.1±0.1	10.1±1.9
**C_5S-Cu NPs**	63.9±2.7	4.0±0.9	2.5±0.5	16.9±2.0	0.1±0.1	9.9±2.5
**C_{12}S-Cu NPs**	7.2±1.9	20.1±2.0	0.0±0.0	8.4±1.1	9.1±1.9	50.9±3.7
**C_{18}S-Cu NPs**	6.1±1.3	20.0±1.8	0.0±0.0	10.3±1.1	7±1.0	58.3±4.1
**C_{22}S-Cu NPs**	49.0±5.1	9.5±2.1	1.5±0.1	5.2±2.6	0.4±0.2	23.6±3.6

Supplementary Table 6. Electrochemical performance table of C_{12}S-CuNPs at various current densities.

**Current density (mA/cm^2)**	**H_2 FE (%)**	**C_2H_4 FE (%)**	**CH_4 FE (%)**	**C_2H_5OH FE (%)**	**C_3H_7OH FE (%)**	**CH_3COO^- FE (%)**
**100**	7.6±2.1	18.7±2.9	0.0±0.0	12.6±1.4	12.2±2.4	45.4±3.3
**200**	7.2±1.9	20.1±2.0	0.0±0.0	8.4±1.1	9.1±1.9	50.9±3.7
**300**	6.6±1.4	22.2±3.1	0.0±0.0	8.8±1.4	3.1±1.0	60.1±4.2
**400**	5.7±1.3	23.7±2.7	0.0±0.0	3.9±1.0	0.6±0.3	67.9±3.5

Supplementary Table 7. Electrochemical performance table of C_{18}S-CuNPs at various current densities.

**Current density (mA/cm^2)**	**H_2 FE (%)**	**C_2H_4 FE (%)**	**CH_4 FE (%)**	**C_2H_5OH FE (%)**	**C_3H_7OH FE (%)**	**CH_3COO^- FE (%)**
**100**	5.6±1.5	19.2±2.5	0.0±0.0	13.4±1.3	8.2±0.9	46.1±3.2
**200**	6.1±1.3	20.0±1.8	0.0±0.0	10.3±1.1	7±1.0	58.3±4.1
**300**	5.1±0.8	20.9±2.4	0.0±0.0	6.9±0.7	0.8±0.8	66.2±5.2
**400**	5.1±0.8	22.1±3.1	0.0±0.0	5.4±0.9	0.9±0.4	69.5±4.8

Supplementary Table 8. The ECSA, contact angle, and operating voltage of different loading of C_{18}S-CuNPs.

Loading	Receding Contact angle (degrees)	Advancing Contact angle (degrees)	ECSA (cm^2_{ECSA})	Voltage vs Ag/AgCl at 100 mA/cm^2 (not IR corrected)
**1 mg**	162.1	157.5	37.6	-1.70
**2 mg**	166.9	158.0	23.8	-1.68
**5 mg**	168.8	163.2	16.7	-1.67
**15 mg**	169	163	15.5	-1.67

Supplementary Table 9. Summary of CO electroreduction to acetate on different catalysts.

Catalyst	$FE_{Acetate}$	*$EE_{Acetate}$	J_{tot} (mA cm^{-2})	E (Full cell or vs RHE)	Ref
**SL-Cu nanoparticles**	**70%**<br>**45%**	 **24%**	**400**<br>**100**	-0.67<br>-0.49/2.3_{FC}	**This work**
Triangular Cu nanosheets	45%		200	-0.75	*Nat Catal* **2,** 423–430 (2019)[4].
Cu-Pd alloy	50-70%<br>20%	17.5%	500<br>100	-1.02/3.5_{Fc}<br>-0.72	*Nat. Catal 22, 1-8 (2022)*[5]
Cu Nanoparticles	25-30%	12.5-15%	140	2.32_{FC}	*Joule* **3,** 240–256 (2019)[6].
Micron-sized Cu	20-24%	7-9%	500	3.20_{FC}	*Nat Catal* **1,** 748–755 (2018)[7].
$Ag_2Cu_2O_3$	25%		400	-0.90	*Energy and Environmental Science* **13,** 2993–3006 (2020)[8].

Supplementary Table 10. Electrochemical performance table for the stability test presented in Figure 4d.

Time (h)	H_2 FE (%)	Ethylene FE (%)	Methane FE (%)	Ethanol FE (%)	Propanol FE (%)	Acetate FE (%)
**5.6**	9.0	24.2	0.0	9.9	3.0	47.3
**20.0**	10.8	22.5	0.1	8.8	4.4	48.6
**48.0**	11.1	23.0	0.0	10.3	5.3	44.9
**68.1**	12.2	22.7	0.0	9.5	4.8	46.3
**92.4**	13.5	20.4	0.0	12.8	3.3	44.4
**100**	13.7	19.9	0.0	12.2	3.5	42.7

Supplementary Table 11. Electrochemical performance table of C_{18}S-CuNPs at various current densities in CO_2RR.

Current density (mA/cm^2)	H_2 FE (%)	Ethylene FE (%)	CO FE (%)	Methane FE (%)	Ethanol FE (%)	Propanol FE (%)	Acetate FE (%)	Formate FE (%)
**100**	7.8±1.7	9.2±0.8	41.1±2.8	0.0±0.0	20.0±1.5	0.0±0.2	3.5±1.1	11.2±1.9
**200**	7.9±1.4	19.0±1.5	34.9±2.7	0.1±0.0	15.4±1.5	7.4±0.6	2.4±1.4	8.0±1.7
**300**	7.5±2.0	32.6±2.6	21.9±1.6	0.4±0.2	21.5±2.3	4.5±0.9	4.9±2.2	5.6±1.4
**400**	22.4±2.4	26.5±2.9	8.7±0.5	2.7±1.0	27.2±1.7	5.0±0.4	8.1±1.6	4.7±1.2

Supplementary Table 12. The CORR performance comparison between Oleylamine-CuNPs and CuNPs catalysts.

Catalyst	H_2 FE (%)	Ethylene FE (%)	Methane FE (%)	Ethanol FE (%)	Propanol FE (%)	Acetate FE (%)
**Oleylamine+Cu NPs**	35.0%±5.5	8.6%±2.3	0.5%±0.2	10.0±2.2	2.1±0.8	16.0±2.3
**Cu NPs**	39.7±2.3	10.8±1.2	0.0±0.0	7.4±1.8	5.4±0.9	13.5±1.9

References

1 Kwok, S. C., Ciucci, F. & Yuen, M. M. Chemisorption threshold of thiol-based monolayer on copper: effect of electric potential and elevated temperature. *Electrochimica Acta* **198**, 185-194 (2016).

2 Zhou, Y. *et al.* Dopant-induced electron localization drives CO2 reduction to C2 hydrocarbons. *Nature chemistry* **10**, 974-980 (2018).

3 Zelinka, S. L., Kirker, G. T., Sterbinsky, G. E. & Bourne, K. J. Oxidation states of copper in preservative treated wood as studied by X-ray absorption near edge spectroscopy (XANES). *Plos one* **17**, e0263073 (2022).

4 Luc, W. *et al.* Two-dimensional copper nanosheets for electrochemical reduction of carbon monoxide to acetate. *Nature Catalysis* **2**, 423-430 (2019).

5 Ji, Y. *et al.* Selective CO-to-acetate electroreduction via intermediate adsorption tuning on ordered Cu–Pd sites. *Nature Catalysis*, 1-8 (2022).

6 Ripatti, D. S., Veltman, T. R. & Kanan, M. W. Carbon monoxide gas diffusion electrolysis that produces concentrated C2 products with high single-pass conversion. *Joule* **3**, 240-256 (2019).

7 Jouny, M., Luc, W. & Jiao, F. High-rate electroreduction of carbon monoxide to multi-carbon products. *Nature Catalysis* **1**, 748-755 (2018).

8 Martić, N. *et al.* Ag 2 Cu 2 O 3–a catalyst template material for selective electroreduction of CO to C 2+ products. *Energy Environmental Science* **13**, 2993-3006 (2020).
