# Supplementary material for: Ligand-modified nanoparticle surfaces influence CO electroreduction selectivity
Source: Nat Commun. 2024 Apr 6;15:2995. doi: 10.1038/s41467-024-47319-z (PMC10998913; doi:10.1038/s41467-024-47319-z)
Supplement: Supplementary file 3 — Description of Additional Supplementary Files [file 41467_2024_47319_MOESM3_ESM.pdf]

Description of Additional Supplementary Files

Supplementary Movie 1. The vibration simulation of the Cu-S bond at 311.3 cm^{-1} on the C_2S-Cu slab from DFT calculations.
Supplementary Movie 2. The vibration simulation of the Cu-CO at 390.3 cm^{-1} on the clean Cu slab from DFT calculations.
Supplementary Movie 3. The vibration simulation of the Cu-CO at 420.5 cm^{-1} on the C_2S-Cu slab from DFT calculations.
Supplementary Movie 4. The vibration simulation of the C-S bond at 618.8 cm^{-1} on the C_2S-Cu slab from DFT calculations.
